# Outcome of endoscopic transsphenoidal surgery for acromegaly: Comparison of using and not using the floor standing pneumatic powered endoscope-holder system

**DOI:** 10.1016/j.heliyon.2024.e35647

**Published:** 2024-08-02

**Authors:** Masahiko Tosaka, Rei Yamaguchi, Kazuhiko Horiguchi, Atsushi Ozawa, Shunichi Matsumoto, Fumiaki Honda, Yohei Hokama, Takaaki Yoshida, Mitsuko Okano, Akihiro Tsukada, Shogo Ishiuchi, Masanobu Yamada, Yuhei Yoshimoto

**Affiliations:** a Department of Neurosurgery, Gunma University Graduate School of Medicine, Maebashi, Gunma, Japan; b Division of Endocrinology and Metabolism, Department of Internal Medicine, Gunma University Graduate School of Medicine, Maebashi, Gunma, Japan; c Department of Neurosurgery, Graduate School of Medicine, University of the Ryukyus, Nishihara, Okinawa, Japan; d Department of Neurosurgery, Saku Central Hospital Advanced Care Center, Saku, Nagano, Japan; e Department of Neurosurgery, Hokushin General Hospital, Nakano, Nagano, Japan

**Keywords:** Acromegaly, Growth hormone, Pituitary neuroendocrine tumor, Endoscopy, Endoscope holder

## Abstract

**Introduction:**

Endoscopic transsphenoidal surgery can be performed by two surgeons, including an endoscopist (PE/2S), and by a single surgeon with an endoscope-holder system (PE/1S + H). We analyzed the surgical outcome, and outcome predictors in acromegaly patients in endoscopic transsphenoidal surgery using floor standing pneumatic endoscope-holder system.

**Methods:**

Endoscopic transsphenoidal surgery was performed with PE/1S+H (n = 51) and PE/2S (n = 20). Postoperative remission was evaluated by the 2010 consensus criteria for acromegaly. We compared the surgical results of PE/2S style and PE/1S+H style, and investigated the factors associated with favorable surgical outcomes.

**Results:**

There was no difference in clinical background between the PE/2S and the PE/1S + H groups. The remission rates for PE/2S and PE/1S+H were 65.0% and 82.4%, respectively, with no significant difference (p = 0.128). In consecutive 71 cases, statistically useful predictors of remission were low preoperative growth hormone (GH) level (<12 ng/mL), low Knosp grade (0–2), and low revised Knosp grade (0–3A). In the conventional Knosp grade 0–2 and 3/4, the sensitivity was 0.76 and the specificity was 0.81. In the revised Knosp grade 0–3A and 3B/4, the sensitivity was 0.96 and the specificity was 0.44.

**Conclusion:**

The outcome of GH-producing pituitary neuroendocrine tumors surgically removed by PE/1S+H could be almost equivalent to that by PE/2S. Preoperative low GH level and Knosp grades, including revised Knosp grades, are useful preoperative predictors for surgical remission of acromegaly.

## 1 Introduction

Acromegaly is a rare disorder, with an estimated incidence of 3.7 per million per year [1]. The vast majority of acromegaly cases are caused by growth hormone (GH)-producing pituitary neuroendocrine tumors. Excessive GH and insulin-like growth factor-1 (IGF-1) secretion causes progressive somatic disfigurement (mainly involving the face and extremities) and various systemic complications such as hypertension, diabetes, myocardial damage, sleep apnea syndrome, and malignant tumors [2].

Transsphenoidal surgery (TSS) is a well-established, safe method for the treatment of pituitary tumors that has been continuously improved over the last 100 years. TSS approaches the pituitary gland from the nasal cavity, and was previously performed by an operating microscope, but endoscopic endonasal surgery, which had been developed by otolaryngologists, was introduced for endonasal TSS as endoscopic transsphenoidal pituitary surgery around 2000 [3,4]. The introduction of the endoscope has brought about major changes in endonasal pituitary and skull base surgery. Endoscopic endonasal procedures require holding of the endoscope in addition to precise two-handed surgery. In addition, TSS requires both deep understanding of the complex intranasal anatomy and experience with neurosurgical pituitary surgery. Therefore, the world standard for endoscopic endonasal pituitary and skull base surgery is performed by 2 surgeons (one of whom is the endoscopist) in collaboration with an otolaryngologist and a neurosurgeon [[3], [4], [5]].

**Fig. 1:**
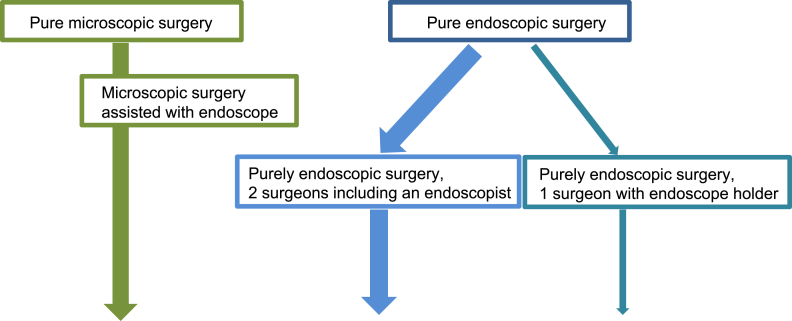
Surgical style development of microscopic and endoscopic pituitary surgery.

An alternative technique in which the endoscope was mechanically fixed and surgery was performed by one surgeon was also considered from the beginning. Initially, a type of endoscope holder was developed that was fixed to the surgical bed. The floor standing pneumatic mechanical endoscope-holder system, EndoArm (Olympus Corp., Tokyo, Japan), was released in 2004 [6,7]. The endoscope and the main body installed on the floor of the operating room are integrated, and can be fixed, released, and moved easily. The UniArm (Mitaka Kohki Co., Ltd., Mitaka, Tokyo, Japan) released in 2008 is basically the same, but the endoscope can be changed universally. Some pioneers of pure endoscopic endonasal pituitary surgery in Japan had already started TSS using these fixation machines as a single surgeon method. These developments led to the emergence of several variations in endonasal pituitary surgery: complete microsurgery, endoscope-assisted microsurgery [8], pure endoscopic surgery with two surgeons, mainly in collaboration with otolaryngologists (PE/2S), pure endoscopic surgery by a single surgeon with the endoscope-holder system (PE/1S+H), and others. The PE/2S style is overwhelmingly used worldwide, with few reports on PE/1S+H (Fig. 1). In particular, there is insufficient data on the outcome of PE/1S+H for GH-producing tumors [9,10].

The present study analyzed surgical outcome, and outcome predictors in 71 patients treated for acromegaly. We compared the surgical results of PE/2S style and PE/1S+H style. In this consecutive 71 cases, we investigated the factors associated with favorable surgical outcomes.

## 2 Methods

### 2.1 Patients

This multicenter retrospective observational study included a series of 71 consecutive patients, 41 females (57.7 %) and 30 males (42.3 %), median age 48.0 (interquartile range, 40–61) years, treated for newly diagnosed GH-producing pituitary tumor from August 2010 to May 2021. A total of 326 pituitary tumor surgeries were performed by the same surgeon (first author) at 2 teaching and tertiary care university hospitals (Gunma University Hospital and Ryukyu University Hospital), which are major referral sites for patients with pituitary tumor, and 2 tertiary care hospitals (Saku Central Hospital Advanced Care Center and Hokushin General Hospital). During this period, the surgeon traveled from main university hospital (Gunma University) to these other hospitals at the time of each surgery to perform endoscopic surgery. This study was reviewed and approved by the institutional review board of Gunma University Graduate School of Medicine (H2020-175), Ryukyu University Graduate School of Medicine (1752), Saku Central Hospital Advanced Care Center (R202012-17), and Hokushin General Hospital (2020022). The institutional review boards approved the opt-out method of informed consent.

### 2.2 Surgical instruments and procedures

**Fig. 2:**
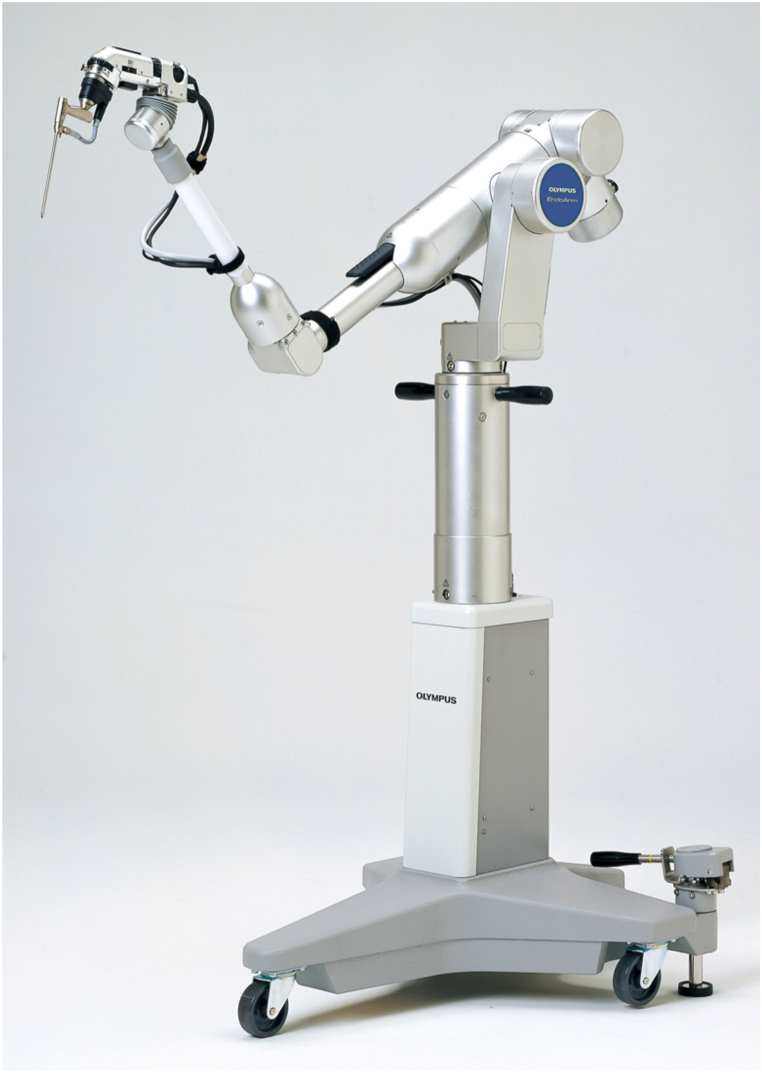
Photo provided by Olympus Corp., Tokyo, Japan. EndoArm, the floor standing pneumatic mechanical endoscope-holder system was released in 2004 from Olympus Corp, Tokyo, Japan.

Purely endoscopic TSS were performed by a main surgeon with an endoscopist before April 2014 (PE/2S, n = 20). Around this time, the EndoArm, a floor-standing pneumatic endoscope holder system, was introduced to our university hospital and other institutions (Fig. 2). Since then, purely endoscopic TSS has been performed with the EndoArm (PE/1S+H, n = 51). The EndoArm incorporating a 4-mm rigid high-definition endoscope was used in 86 % of all surgeries with an endoscope fixing arm [11]. A combination of a 4-mm rigid endoscope (Karl Storz Endoscopy, Tuttlingen, Germany) and the UniArm was used in the other 14 % of surgeries. The endoscope was held with the left hand and surgical manipulations mainly with one hand in the nasal phase. The endoscope was then attached to the floor standing pneumatic powered endoscope-holder system. The procedure was performed using a 0° straight scope, but sometimes a 30° scope was also used. The approach was through both nostrils in all cases. No self-irrigating system was used for the endoscope. A suction tube with a water injection device was used to clean the lenses. In general, the tumor is difficult to approach in patients with acromegaly [5]. Vasoconstricting agents were adequately used for mucosal edema. Anterior sphenoidotomy was performed widely in all cases. The middle nasal turbinate was not resected in this series. The strategy of the approach was the same for PE/2S or PE/1S+H. Tumor removal was based on the extracapsular removal technique. The wall of the cavernous sinus was not resected in all cases, and any cavernous sinus invasion was treated by resection of the inside of the cavernous sinus using collapse of the medial wall by the tumor invasion site. In the usual transsellar approaches, any intraoperative cerebrospinal fluid leak was corrected by filling the sella with fat from the abdomen and the dura was sutured with a few stitches. Extended TSS was also applied for ectopic pituitary tumor of the suprasellar subarachnoid space, according to the transtubercular transplanum approach [12]. In extended TSS, closure was achieved with a fascia patchwork closure with deep suturing [13]. There were no cases in which a nasoseptal flap was elevated.

### 2.3 Radiological analysis

The size and extension of the tumor as well as the degree of resection were assessed by pre- and postoperative T1-weighted magnetic resonance (MR) imaging after injection of gadolinium. Postoperative imaging was performed within 1 month after surgery. Tumor sizes were measured, and classified as microadenoma (<1 cm in maximum diameter) or macroadenoma (≥1 cm in maximum diameter) on any plane. Cavernous sinus invasion was classified according to the Knosp classification [14], which is based on lateral extension of the tumor on the coronal MR images with the internal carotid artery (ICA) serving as the radiological landmark [15]. The Knosp classification consists of five grades [15]. The highest grade of parasellar extension on both sides determines the grade assignment. The conventional Knosp grade was modified with subclassification of Knosp grade 3 in 2015, called the revised Knosp classification [16]: Grade 3A, extension of the tumor into the superior cavernous sinus compartment beyond the lateral border of the ICA but without total encasement of the intracavernous ICA; and grade 3B, involvement of the inferior cavernous sinus compartment beyond the lateral borders of the intra- and supracavernous ICA but without total encasement of the intracavernous ICA [15].

### 2.4 Endocrine analysis

Patients underwent preoperative endocrinological evaluation of fasting GH and IGF-1 levels, and anterior pituitary function. Oral glucose tolerance test (OGTT) was also preoperatively performed in all patients without diabetes. Hormone replacement therapy was administered, if necessary, to patients with clinical and laboratory evidence of hypopituitarism. The 2010 remission criteria consisted of 1) random GH < 1.0 mg/L or nadir GH < 0.4 mg/L after the OGTT, and 2) normalization of age- and sex-adjusted IGF-1. OGTT was not performed in every patient but was performed for equivocal cases with normal IGF-1 and GH > 1.0 mg/L [[17], [18], [19], [20]]. All but 2 patients achieved the remission criteria by 1 year after surgery. The other 2 patients reached the remission criteria by 1.8 year after surgery without further treatment, and were included in the remission group. All patients requiring additional treatment such as medical treatment, reoperation, and gamma knife treatment were included in the non-remission group.

### 2.5 Statistical analysis

Data were analyzed with commercial software SPSS for Mac OS (version 24.0, IBM Corp.) All variables are shown as median (interquartile range) or actual numbers (percentages). The relationships between PE/2S group and PE/1S+H group, and between remission and various clinical factors were examined by univariate analyses using Fisher's accurate probability test and Mann-Whitney *U* test. The analysis included the two Knosp grade classifications, conventional Knosp grade (0–2 vs. 3/4) and revised Knosp grade (0–3A vs. 3B/4). Multiple comparison tests were performed with a Bonferroni correction. A value of p < 0.05 was considered to be statistically significant.

## 3 Results

### 3.1 Patient characteristics

The clinical characteristics of the 71 patients who received purely endoscopic surgery for GH-producing pituitary tumor are shown in Table 1. The former 20 (28.2 %) surgeries were performed without the endoscope fixing arm. The endoscopist held the endoscope for the PE/2S style. More recent 51 (71.8 %) surgeries were performed with the floor standing pneumatic powered endoscope-holder system (EndoArm or UniArm). The remission criteria were achieved in 53 patients up to 1 year after surgery, and in 2 patients within 1.8 years after surgery.

**Table 1:** Characteristics of 71 patients with GH-producing pituitary neuroendocrine tumor.

Sex, no. (%)	
Female	41 (57.7)
Male	30 (42.3)
Age, median (IQR), years	48.0 (40–61)
Type, no. (%)	
Microadenoma	10 (14.1)
Macroadenoma	61 (85.9)
Tumor size, median (IQR), mm	16.8 (12.5–24.2)
Preoperative GH level, median (IQR), ng/mL	12.8 (5.5–29.6)
Preoperative IGF-1 level, median (IQR), ng/mL	575 (455–837)
Visual field defect, no. (%)	
Yes	16 (22.5)
No	55 (77.5)
Knosp grade, no. (%)	
0	16 (22.5)
1	19 (26.8)
2	10 (14.1)
3	21 (29.6)
4	5 (7.0)
Revised Knosp grade, no. (%)	
3A	17 (23.9)
3B	4 (5.6)
Endoscope, no. (%)	
Without mechanical holder	20 (28.2)
With mechanical holder	51 (71.8)
Outcome, no. (%)	
Remission	55 (77.5)
Non-remission	16 (22.5)

IQR = interquartile range.

### 3.2 Comparison between PE/2S group and PE/1S+H group patients

We compared 20 patients in the PE/2S group and 51 patients in the PE/1S+H group (Table 2). Continuous values (age, tumor size, preoperative GH level, preoperative IGF-1 level) were dichotomized by selecting an appropriate standard value with reference to each median value. The proportion of women was higher in the PE/2S group (p = 0.031). There were no differences between the two groups in other factors (age, tumor type, tumor size, preoperative GH level, preoperative IGF-1 level, visual disturbance, Knosp grade, revised Knosp grade). The remission rates for PE/2S and PE/1S+H were 65.0% and 82.4%, respectively. There were also no differences in remission rate (p = 0.128), and achievement of gross total removal on postoperative MR imaging (p = 0.491).

**Table 2:** Comparison between PE/2S group and PE/1S+H group patients.

Variables	Total	ETSS with/without mechanical holder		p Value
		with (n = 51)	without (n = 20)	
Sex				
Female	41 (57.7)	25 (49.0)	16 (80.0)	0.031
Male	30 (42.3)	26 (51.0)	4 (20.0)	
Age				0.43
<48 yrs	35 (49.3)	27 (52.9)	8 (40.0)	
≥48 yrs	36 (50.7)	24 (47.1)	12 (60.0)	
Type				0.131
Microadenoma	10 (14.0)	5 (9.8)	5 (25.0)	
Macroadenoma	61 (86.0)	46 (90.2)	15 (75.0)	
Tumor size				0.797
<17 mm	37 (52.1)	26 (51.0)	11 (55.0)	
≥17 mm	34 (47.9)	25 (49.0)	9 (45.0)	
Preoperative GH level				0.43
<12 ng/mL	35 (49.3)	27 (52.9)	8 (4.0)	
≥12 ng/mL	36 (50.7)	24 (47.1)	12 (60.0)	
Preoperative IGF-1 level				0.598
<580 ng/mL	37 (52.1)	28 (54.9)	9 (45.0)	
≥580 ng/mL	34 (47.9)	23 (45.1)	11 (55.0)	
Visual disturbance				0.76
Yes	16 (22.5)	11 (21.6)	5 (25.0)	
No	55 (77.5)	40 (78.4)	15 (75.0)	
Knosp grade				1.00
0–2	45 (63.4)	32 (62.7)	13 (65.0)	
3 and 4	26 (36.6)	19 (37.3)	7 (35.0)	
Revised Knosp grade				1.00
0–3A	62 (85.9)	44 (86.3)	18 (90.0)	
3B and 4	9 (14.1)	7 (13.7)	2 (10.0)	
Remission				0.128
Yes	55 (77.5)	42 (82.4)	13 (65.0)	
No	16 (22.5)	9 (17.6)	7 (35.0)	
Postoperative MR imaging				0.491
Gross total removal	60 (84.5)	44 (86.3)	16 (80.0)	
Residual	11 (15.5)	7 (13.7)	4 (20.0)	
Operation time, median (IQR)	325 (123)	322 (124)	353 (134)	0.227
Blood loss, median (IQR)	416 (420)	450 (380)	355 (483)	0.190

Values are expressed as number (%) of patients unless otherwise indicated. IQR = interquartile range.

### 3.3 Outcome predictors in acromegaly patients

The outcome predictors of continuous 71 GH-producing tumor patients who received purely endoscopic endonasal surgery are shown in Table 3. Continuous values (age, tumor size, preoperative GH level, preoperative IGF-1 level) were dichotomized by selecting an appropriate standard value with reference to each median value. Lower preoperative GH level (<12 ng/mL) was significantly associated with remission than higher preoperative GH level (≥12 ng/mL) (p < 0.001). Remissions were often achieved with preoperative Knosp grades 0–2, but less likely with Knosp grades 3 and 4 (p < 0.001). Endoscopic cavernous sinus invasion was considered to be absent in 3/4 of revised Knosp grade 3A cases [15]. Therefore, revised Knosp grade 3A was classified in the lower Knosp grade, and grade 3B in the higher Knosp grade. Remissions were often achieved with the preoperative lower revised Knosp grade, but less likely with the higher revised Knosp grade (p < 0.001). Achievement of gross total removal on postoperative MR imaging was one of the criteria for postoperative remission (p < 0.001).

**Table 3:** Outcome predictors in 71 patients with GH-producing pituitary neuroendocrine tumor.

Variables	Total	Remission		p Value
		Yes (n = 55)	No (n = 16)	
Sex				
Female	41 (57.7)	30 (54.5)	11 (68.8)	0.395
Male	30 (42.3)	25 (45.5)	5 (31.3)	
Age				0.267
<48 yrs	35 (49.3)	25 (45.5)	10 (62.5)	
≥48 yrs	36 (50.7)	30 (54.5)	6 (37.5)	
Type				0.103
Microadenoma	10 (14.1)	10 (18.2)	0 (0.0)	
Macroadenoma	61 (85.9)	45 (81.8)	16 (100.0)	
Tumor size				0.022
<17 mm	37 (52.1)	33 (60.0)	4 (25.0)	
≥17 mm	34 (47.9)	22 (40.0)	12 (75.0)	
Preoperative GH level				<0.001^a^
<12 ng/mL	35 (49.3)	34 (61.8)	1 (6.3)	
≥12 ng/mL	36 (50.7)	21 (38.2)	15 (93.7)	
Preoperative IGF-1 level				0.257
<580 ng/mL	37 (52.1)	31 (56.4)	6 (37.5)	
≥580 ng/mL	34 (47.9)	24 (43.6)	10 (62.5)	
Visual disturbance				0.038
Yes	16 (22.5)	9 (16.4)	7 (43.8)	
No	55 (77.5)	46 (83.6)	9 (56.3)	
Knosp grade				<0.001^a^
0–2	45 (63.4)	42 (76.4)	3 (18.8)	
3 and 4	26 (36.6)	13 (23.6)	13 (81.3)	
Revised Knosp grade				<0.001^a^
0–3A	62 (87.3)	53 (96.4)	9 (56.3)	
3B and 4	9 (12.7)	2 (3.6)	7 (43.8)	
Endoscope				0.128
With mechanical holder	51 (71.8)	42 (76.4)	9 (56.3)	
Without mechanical holder	20 (28.2)	13 (23.6)	7 (43.8)	
Postoperative MR imaging				<0.001^a^
Gross total removal	60 (84.5)	55 (100)	5 (31.3)	
Residual	11 (16.9)	0 (0)	11 (68.8)	

Values are expressed as number (%) of patients unless otherwise indicated.

a p < 0.001.

Our series included 26 cases of high Knosp grade (3 and 4) tumor. In this study, we revised the low Knosp grade to include Knosp grade 3A for our analysis [15,16]. Remission was achieved in 11 of 17 patients with revised Knosp grade 3A, 2 of 4 with grade 3B, and 0 of 5 with grade 4. The relationship between remission and grade was investigated by comparing the conventional Knosp grade division between grade 0–2 and grade 3/4, and the revised Knosp grade division between grade 0–3A and grades 3B/4. In the conventional Knosp grade 3 and 4, the sensitivity was 0.76, and the specificity was 0.81. In the revised Knosp grade 0–3A and 3B/4, the sensitivity was 0.96, and the specificity was 0.44. We found that our new classification using the revised Knosp grade increases sensitivity but decreases specificity.

**Fig. 3:**
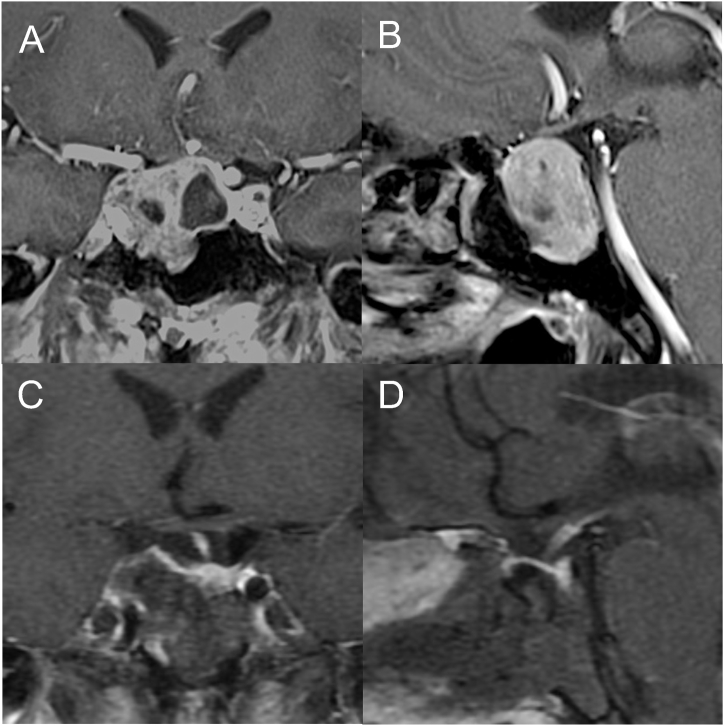
Representative Case 1: A 44-year-old man with GH-producing pituitary neuroendocrine tumor. The preoperative values were as high as 25.6 ng/mL for GH, 86.3 ng/mL for prolactin, and 805 ng/mL for IGF-1 (standard range: 92–255 ng/mL). Preoperative coronal (A) and sagittal (B) T1-weighted MR images after injection of gadolinium showing a tumor with maximum diameter of 29.0 mm and lateral extension of Knosp grade 3A to the right. The normal pituitary gland was on the left side. Postoperative coronal (C) and sagittal (D) T1-weighted MR images after injection of gadolinium showing complete removal of the tumor. Postoperative GH level was 0.72 ng/mL, 3.2 ng/mL for prolactin, and IGF-1 level was 239 ng/mL, showing remission without affect on other pituitary hormones after surgery.

**Fig. 4:**
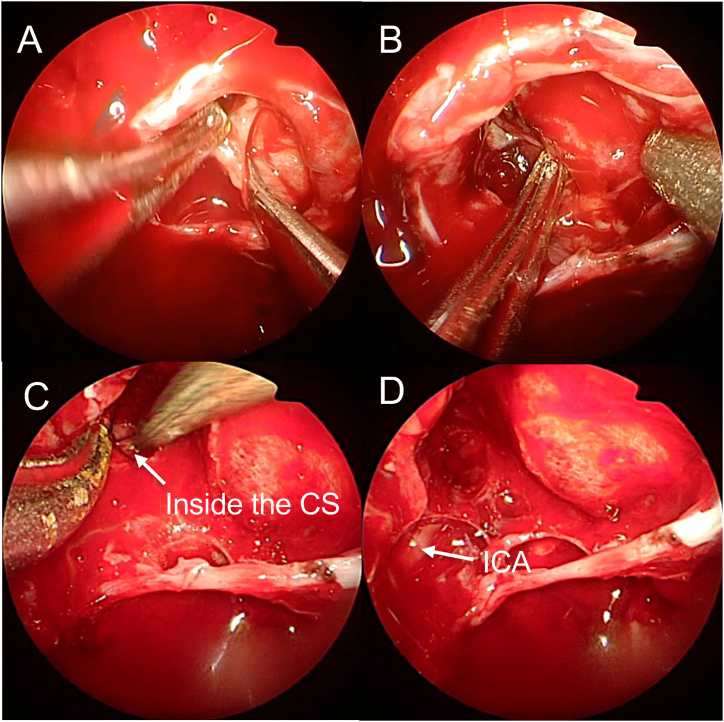
Intraoperative endoscopic pictures of the representative Case 1: (A) Extracapsular dissection on the normal pituitary side. (B) Tumor is largely removed by suction. (C) Tumor inside the cavernous sinus side (CS, arrow). (D) Medial wall of the cavernous sinus after removal of the tumor. Arrow indicates the internal carotid artery (ICA).

**Fig. 5:**
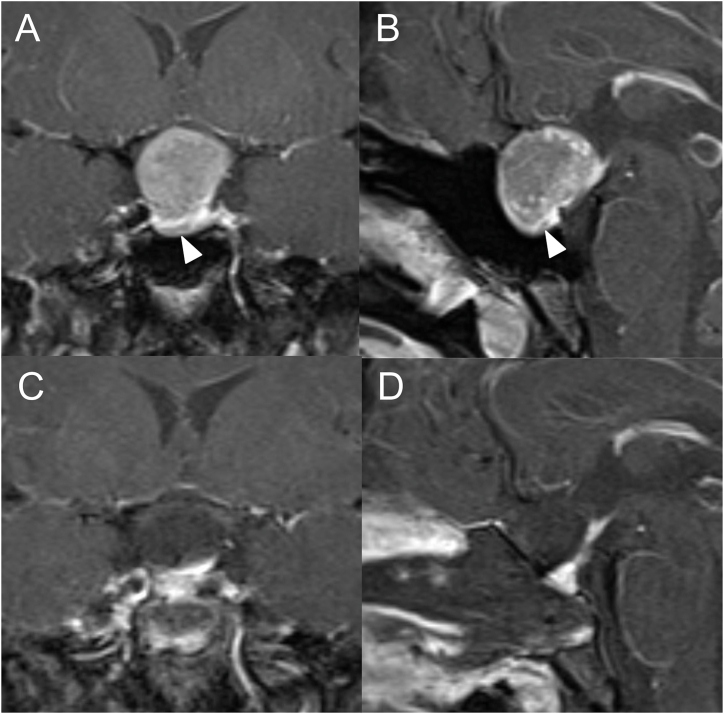
Representative Case 2: A 25-year-old woman with ectopic suprasellar GH-producing pituitary neuroendocrine tumor. Preoperative GH level was 17.41 ng/mL and IGF-1 level was 725 ng/mL (standard range: 147–358 ng/mL). Preoperative coronal (A) and sagittal (B) T1-weighted MR images after injection of gadolinium showing a tumor with maximum diameter of 20.2 mm, the normal pituitary gland below the tumor, and bottom of the sella (arrowhead). Postoperative coronal (C) and sagittal (D) T1-weighted MR images after injection of gadolinium showing complete removal of the tumor. Basal GH level was 0.38 ng/mL and IGF-1 level was 172 ng/mL, and remission was achieved after surgery.

**Fig. 6:**
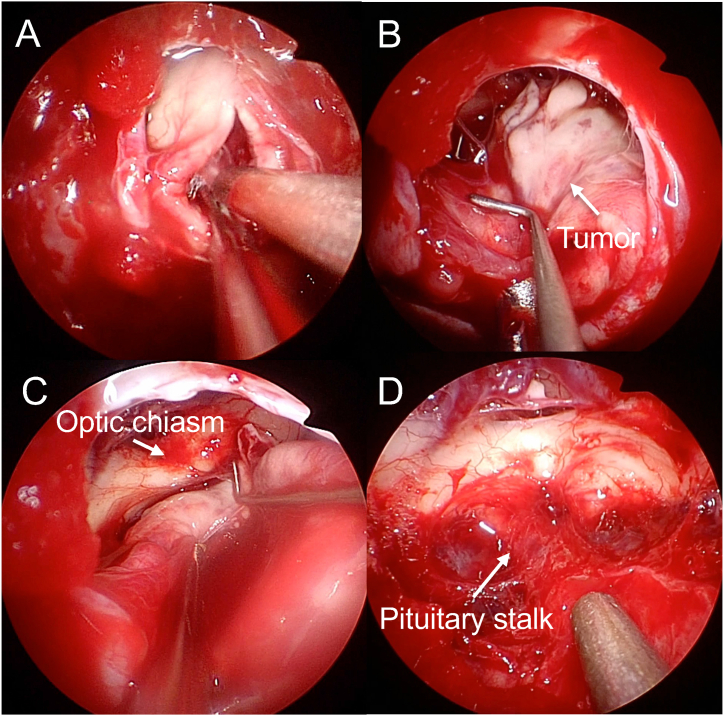
Intraoperative endoscopic pictures of the representative Case 2. The ectopic pituitary neuroendocrine tumor was located in the suprasellar subarachnoid space. (A) Tumor was internally decompressed in the capsule. (B) Observation of the right side of the tumor (arrow). (C) Dissection of the tumor from the optic chiasm (arrow). (D) After total removal. Pituitary stalk was observed (arrow).

We provide endoscopic intraoperative images from representative cases for demonstration of endoscopic TSS for acromegaly using floor standing pneumatic powered endoscope-holder system (Fig. 3[A–D], Fig. 4[A–D], Fig. 5[A–D], Fig. 6[A–D]).

### 3.4 Complications

The operative time for the PE/2S and PE/1S+H methods was median 353 (interquartile range [IQR] 134) and 322 (124) minutes, respectively (P = 0.227). The principal surgeon was also a technical instructor, and the operative time was always extended to provide guidance to younger neurosurgeons and residents. The estimated blood loss in the PE/2S and PE/1S+H methods was 355 (483) and 450 (380) mL, respectively (P = 0.19). No transfusions other than prepared autologous blood were performed except in one case (280 mL). General anesthesia was performed by preparing an endoscope for intubation and frequently using the awake intubation technique. No intraoperative risks or events such as reintubation after extubation occurred. Uneventful management of general anesthesia was performed in all cases.

Transient or permanent postoperative hormone supplements were administered in 7 patients. Six patients required permanent anterior pituitary hormone replacement, but 3 of these 6 had preoperative anterior pituitary dysfunction. All three patients who required new hormone replacement were included in the PE/1S+H group. No permanent diabetes insipidus was observed, but transient diabetes insipidus was observed in 13 patients (21 %) (PE/2S: PE/1S+H = 8:5, not significant). Transient hyponatremia occurred in 10 patients (14.1 %) and was successfully treated with fluid restriction (PE/2S: PE/1S+H = 5:5, not significant). A patient with postoperative nasal bleeding required hemostatic treatment. No cases of mild or severe sinusitis required treatment with postoperative antibiotics (other than postoperative prophylactic antibiotics). No major complications, including major morbidity, mortality, cerebrospinal fluid leakage, meningitis, and intracerebral hemorrhage, occurred in this series. No major intraoperative complications, including internal carotid artery injury, or problems related to general anesthesia occurred. All 16 patients (22.5 %) with preoperative visual acuity and field deficits improved after surgery without new visual deficits.

## 4 Discussion

### 4.1 History of endoscope holding styles in pure endoscopic pituitary surgery

TSS for pituitary tumors was originally established as pure endoscopic surgery in 1996 [3,4]. Since then, the introduction of endoscopic surgery in this field has steadily expanded, and now many operations are performed under an endoscope, whereas the use of the operating microscope has decreased significantly [21]. There are two styles of endoscopic surgery, pure endoscopic pituitary surgery with two surgeons (PE/2S) [3,4,7,17,22,23], requiring the endoscopist to hold the endoscope, and pure endoscopic surgery with a single surgeon using the endoscope-holder system (PE/1S+H) [6,9,10].

Development of the mechanical arm for holding the endoscope in PE/1S+H has mainly occurred in the field of otolaryngology since the very early days [24]. The Unitrac® Pneumatic Holding Arm (B. Braun Aesculap Japan Co., Ltd., Tokyo, Japan) is available as a pneumatic holder system fixed to the operating table [9]. However, surgery using a mechanical holder involves greater interference due to the completely fixed shaft of the endoscope in a narrow nasal cavity. Therefore, the surgeon required a learning curve at the time of introduction of the mechanical holder system, preventing widespread adoption [6,7,9,10]. Consequently, surgery performed by the endoscopist holding the endoscope (PE/2S) became mainstream worldwide [3,4,17,22,23].

In contrast, the floor standing mechanical endoscope-holder system was released in 2004 and has become widespread in Japan [10,11]. This product was originally designed for surgery for various intracranial lesions, including hematoma, aneurysm, and skull base lesions. Some pituitary surgeons also use floor standing mechanical endoscope-holder systems [[9], [10], [11], [12], [13]]. However, the outcome of endoscopic surgery using mechanical endoscope holders has not been well reported. Because the PE/1S+H style using floor standing mechanical endoscope-holder system is still a minor method worldwide (Fig. 1). Outcomes of single surgeon surgery on 300 different cases using the UniArm were reported [10]. The remission rate was 72 % for 41 GH-producing tumor cases in this series. However, the remission criteria were those defined in 2003, which are more lenient than the current 2010 criteria, so the remission rate using the current criteria is unknown [10]. The present study compared outcomes with PE/1S+H (51 cases) with PE/2S (20 cases) under the current 2010 criteria for GH-producing pituitary tumor. The remission rates for PE/2S and PE/1S+H were statistically equivalent at 65.0 % and 82.4 %, respectively (77.5 % of all 71 cases). This result may become the benchmark for the outcome of single surgeon-style pituitary surgery with a floor standing pneumatic endoscope holder for GH-producing tumors under the current remission criteria.

### 4.2 Comparison between PE/2S style and PE/1S+H style

Surgery using the PE/1S+H style has several disadvantages compared to surgery using the PE/2S style (Table 4). First, the PE/1S + H style causes considerable interference because the endoscope is mechanically fixed. Consequently, the surgeon must overcome the learning curve before achieving a high degree of freedom in surgery. Second, PE/2S style allows the observer (surgeon) to easily obtain a pseudo-three-dimensional impression because the free endoscope allows slight and continuous movement. In contrast, the PE/1S+H style only allows a two-dimensional image, and so any three-dimensional impression is difficult to obtain. Three-dimensional endoscopes are commercially available, but are not widely used due to the drawbacks such as the thicker endoscope. Third, the scopist can instantly bring the endoscope closer to the focal point to zoom on the screen, and a magnified intraoperative image can be easily and continuously obtained, which is another great advantage of PE/2S. The PE/1S+H style is difficult to zoom in instantly, and training is required to enable surgery with a magnified image.

**Table 4:** Operation styles of endoscopic endonasal surgery for GH-producing pituitary neuroendocrine tumor.

	PE/2S	PE/1S+H
Surgeon	Two surgeons, including a scopist	One surgeon
Advantage	1) 3D feeling by movement of endoscope 2) Scopist can easily create the close field of view 3) Three or four hands technique 4) Consultation for intraoperative decision making	1) Fine manipulation 2) Need only 1 surgeon
Disadvantage	1) Need 2 surgeons 2) Interference in 2 surgeons hands 3) Small movement of the endoscope	1) Need endoscope holder 2) Complete 2D view (3D endoscope is available) 3) Strong interference and learning curve

PE/2S = Pure endoscopic surgery with two surgeons, including a scopist; PE/1S+H = Pure endoscopic surgery with a surgeon with an endoscope-holder system; 3D = three-dimensional; 2D = two-dimensional.

Despite these major drawbacks, some surgeons prefer the PE/1S+H style as complete fixation of the endoscope allows finer manipulation, and precise manipulation is possible. Repositioning of the scope with the pneumatic endoscope holder provides more than adequate grasp of the three-dimensional anatomy, and the depth of field afforded by endoscopes typically more than compensates for any lack of stereopsis [10]. Furthermore, we believe that even with a fixed endoscope, a three-dimensional image is formed in the surgeon's brain by combining the movement of the suction tube and forceps on the two-dimensional monitor with the depth sense of the hand that manipulates them. In the PE/2S method, the suction tube and forceps move under a moving endoscope. Such moving field of view slightly reduces the visual accuracy of fine instrument movements. Therefore, a fixed view (PE/1S+H) may allow finer and more precise manipulations. For example, deep suturing at the base of the skull through the endonasal route, which requires very fine control of the needle tip, was initially developed under endoscopic fixation using a holder [13].

Another advantage is that only one surgeon can perform the surgery. Consequently, the same surgeon can easily move and perform surgeries at any multiple centers if the holder system is available. Final determination of which of these two methods is better will be difficult. This choice may also depend on the surgeon's preference and comfort.

Of course, the PE/2S style can also be performed very finely by a skillful surgeon. In this study, the remission rate of GH-producing tumor treated by PE/1S+H was not inferior to that by PE/2S. The method of mechanically fixing the endoscope needs to be tested and evaluated by more pituitary surgeons worldwide.

### 4.3 Surgical outcome predictors in acromegaly patients

Surgical outcomes for GH-producing pituitary tumors have been compared between microsurgery and endoscopic surgery [23,25,26]. However, endoscopic surgery did not appear superior to microsurgery [23,25,26]. The remission rate for endoscopic surgery for GH-producing pituitary tumors using the current 2010 criteria was 45–71 % in a relatively large series of more than 40 patients [22]. The remission rate for the entire series was 77.5 % in the present study, equivalent to the previous reports [22].

Preoperative clinical factors that predicted the remission of GH-producing pituitary tumor include preoperative GH level, IGF-1 level, tumor size, primary or reoperation, age, and higher Knosp grade [22,25]. In particular, Knosp grade is an important factor repeatedly identified in many studies [22]. In our study, the significant univariate predictors of remission were low preoperative GH level (<12 ng/mL) and low Knosp grade (0–2). These results were similar to previous reports.

In addition, we evaluated the revised Knosp grade, which divides extension to the lateral side of the ICA into grade 3A and 3B [14,15]. Actual cavernous sinus infiltration was reported to be 26.5 % for grade 3A and 70.6 % for grade 3B [15]. Gross total removal rate and endocrine remission were good in grade 3A and bad in grades 3B and 4 in a series of non-functioning and functioning tumor (23 cases) including 10 cases of GH-producing tumor [27]. However, remission was not achieved in any cases of functional tumors in grade 3A, which is currently controversial [16]. We found that the outcome of grade 3A cases tended to be good, so we added grade 3A to the low conventional Knosp grade group (grades 0–2), compared to the high Knosp grade group (grades 3B and 4). This new division using revised Knosp grade was also associated with postoperative remission of GH-producing tumor. This study found that classification using the revised Knosp grade significantly improved sensitivity compared to classification using conventional Knosp grade. However, specificity was inferior to conventional methods.

### 4.4 Limitations

The limitations of this study include the following. First, the relatively small number of patients may have failed to detect significant statistical differences in outcomes. However, this series does not include so small a number of patients as previous reports of GH-producing tumor surgery. Second, more than double the patients underwent the PE/1S+H technique compared to the PE/2S technique cohort. Therefore, any comparison of numbers may be statistically unbalanced. Third, this series did not include reoperation or recurrence cases. Fourth, long-term follow up will be necessary to determine the long-term remission rates. Fifth, historical bias, or the bias due to the accumulation of the operator's experience, may be related to outcome over a long-term study period. Sixth, this study did not analyze the characteristics of the nasal cavity or evaluate which technique is more suitable. Postoperative mucosal damage in the nasal cavity was also not examined [5,7]. These issues will need to be investigated in the future. Seventh, there is the limitation of the lack of previous studies on the subject of endoscope holders.

## 5 Conclusion

The outcome of endoscopic surgery with mechanically fixing endoscope system (PE/1S+H) may be not inferior than two surgeon style endoscopic surgery (PE/2S) for acromegaly. Preoperative GH level and Knosp grades, including revised Knosp grades, are useful preoperative predictors for surgical remission of GH-producing pituitary tumor.

## Funding statement

This research did not receive any specific grant from funding agencies in the public, commercial, or not-for-profit sectors.

## Data availability statement

Data included in article/supp. material/referenced in article.

## CRediT authorship contribution statement

**Masahiko Tosaka:** Writing – review & editing, Writing – original draft, Project administration, Methodology, Formal analysis, Data curation, Conceptualization. **Rei Yamaguchi:** Writing – review & editing, Validation, Data curation. **Kazuhiko Horiguchi:** Validation, Data curation. **Atsushi Ozawa:** Validation, Data curation. **Shunichi Matsumoto:** Validation, Data curation. **Fumiaki Honda:** Validation, Data curation. **Yohei Hokama:** Validation, Data curation. **Takaaki Yoshida:** Validation, Data curation. **Mitsuko Okano:** Validation, Data curation. **Akihiro Tsukada:** Validation, Data curation. **Shogo Ishiuchi:** Writing – review & editing, Supervision. **Masanobu Yamada:** Writing – review & editing, Supervision. **Yuhei Yoshimoto:** Writing – review & editing, Supervision.

## Declaration of competing interest

The authors declare that they have no known competing financial interests or personal relationships that could have appeared to influence the work reported in this paper.

## References

[1] Arnardóttir S., Järås J., Burman P. (2022). Long-term outcomes of patients with acromegaly: a report from the Swedish Pituitary Register. Eur. J. Endocrinol..

[2] Demir A.N., Sulu C., Kara Z. (2023). Changing presentation of acromegaly in half a century: a single-center experience. Pituitary.

[3] Carrau R.L., Jho H.D., Ko Y. (1996). Transnasal-trans-sphenoidal endoscopic surgery of the pituitary gland. Laryngoscope.

[4] Jho H.D., Carrau R.L. (1997). Endoscopic endonasal transsphenoidal surgery: experience with 50 patients. J. Neurosurg..

[5] Zada G., Cavallo L.M., Esposito F. (2010). Transsphenoidal surgery in patients with acromegaly: operative strategies for overcoming technically challenging anatomical variations. Neurosurg. Focus.

[6] Eskandari R., Amini A., Yonemura K.S., Couldwell W.T. (2008). The use of the Olympus EndoArm for spinal and skull-based transsphenoidal neurosurgery. Minim. Invasive Neurosurg..

[7] Ismail M., Abdelaziz A.A., Darwish M. (2019). A comparison between collaborative and single surgeon approach in endoscopic endonasal surgery to sphenoid sinus. Eur. Arch. Oto-Rhino-Laryngol..

[8] Rennert R.C., Fredrickson V.L., Couldwell W.T. (2022). Microscopic transsphenoidal surgery in the era of endoscopy: are there any advantages?. Otolaryngol Clin North Am.

[9] Yano S., Kawano T., Kudo M. (2009). Endoscopic endonasal transsphenoidal approach through the bilateral nostrils for pituitary adenomas. Neurol. Med.-Chir..

[10] Mamelak A.N., Carmichael J., Bonert V.H., Cooper O., Melmed S. (2013). Single-surgeon fully endoscopic endonasal transsphenoidal surgery: outcomes in three-hundred consecutive cases. Pituitary.

[11] Morita A., Okada Y., Kitano M., Hori T., Taneda M., Kirino T. (2004). Development of hybrid integrated endoscope-holder system for endoscopic microneurosurgery. Neurosurgery.

[12] Miyagishima T., Tosaka M., Yamaguchi R. (2019). Extended endoscopic endonasal resection of craniopharyngioma using intraoperative visual evoked potential monitoring: technical note. Acta Neurochir..

[13] Tosaka M., Prevedello D.M., Yamaguchi R. (2021). Single-layer fascia patchwork closure for the extended endoscopic transsphenoidal transtuberculum transplanum approach: deep suturing technique and preliminary results. World Neurosurg.

[14] Knosp E., Steiner E., Kitz K., Matula C. (1993). Pituitary adenomas with invasion of the cavernous sinus space: a magnetic resonance imaging classification compared with surgical findings. Neurosurgery.

[15] Micko A.S.G., Wöhrer A., Wolfsberger S., Knosp E. (2015). Invasion of the cavernous sinus space in pituitary adenomas: endoscopic verification and its correlation with an MRI-based classification. J. Neurosurg..

[16] Buchy M., Lapras V., Rabilloud M. (2019). Predicting early post-operative remission in pituitary adenomas: evaluation of the modified knosp classification. Pituitary.

[17] Jane JA Jr, Starke R.M., Elzoghby M.A. (2011). Endoscopic transsphenoidal surgery for acromegaly: remission using modern criteria, complications, and predictors of outcome. J. Clin. Endocrinol. Metab..

[18] Giustina A., Chanson P., Bronstein M.D. (2010). Acromegaly Consensus Group. A consensus on criteria for cure of acromegaly. J. Clin. Endocrinol. Metab..

[19] Asha M.J., Takami H., Velasquez C. (2019 Oct 11). Long-term outcomes of transsphenoidal surgery for management of growth hormone-secreting adenomas: single-center results. J. Neurosurg..

[20] Coopmans E.C., Postma M.R., Wolters T.L.C. (2021). Predictors for remission after transsphenoidal surgery in acromegaly: a Dutch multicenter study. J. Clin. Endocrinol. Metab..

[21] Hattori Y., Tahara S., Aso S. (2020). Pituitary surgery's epidemiology using a national inpatient database in Japan. Acta Neurochir..

[22] Taghvaei M., Sadrehosseini S.M., Ardakani J.B., Naåkhjavani M., Zeinalizadeh M. (2018). Endoscopic endonasal approach to the growth hormone-secreting pituitary adenomas: endocrinologic outcome in 68 patients. World Neurosurg.

[23] Guinto G., Guinto-Nishimura G.Y., Uribe-Pacheco R. (2024). Surgical outcomes in patients with acromegaly: microscopic vs endoscopic transsphenoidal surgery. Best Pract. Res. Clin. Endocrinol. Metabol..

[24] Pangal D.J., Cote D.J., Ruzevick J. (2022). Robotic and robot-assisted skull base neurosurgery: systematic review of current applications and future directions. Neurosurg. Focus.

[25] Phan K., Xu J., Reddy R., Kalakoti P., Nanda A., Fairhall J. (2017). Endoscopic endonasal versus microsurgical transsphenoidal approach for growth hormone-secreting pituitary adenomas-systematic review and meta-analysis. World Neurosurg.

[26] Starke R.M., Raper D.M., Payne S.C., Vance M.L., Oldfield E.H., Jane J.A. (2017). Endoscopic vs microsurgical transsphenoidal surgery for acromegaly: outcomes in a concurrent series of patients using modern criteria for remission. J. Clin. Endocrinol. Metab..

[27] Micko A., Oberndorfer J., Weninger W.J. (2019). Challenging Knosp high-grade pituitary adenomas. J. Neurosurg..

